# Cost Evaluation of the Ontario Virtual Urgent Care Pilot Program: Population-Based, Matched Cohort Study

**DOI:** 10.2196/50483

**Published:** 2024-07-15

**Authors:** Jean-Eric Tarride, Justin N Hall, Shawn Mondoux, Katie N Dainty, Joy McCarron, J Michael Paterson, Lesley Plumptre, Emily Borgundvaag, Howard Ovens, Shelley L McLeod

**Affiliations:** 1 Department of Health Research Methods, Evidence and Impact McMaster University Hamilton, ON Canada; 2 Centre for Health Economics and Policy Analysis McMaster University Hamilton, ON Canada; 3 Programs for Assessment of Technology in Health The Research Institute of St. Joe’s Hamilton St. Joseph’s Healthcare Hamilton Hamilton, ON Canada; 4 Department of Emergency Medicine Sunnybrook Health Sciences Centre Toronto, ON Canada; 5 Division of Emergency Medicine Department of Medicine, Temerty Faculty of Medicine University of Toronto Toronto, ON Canada; 6 Institute of Health Policy, Management and Evaluation University of Toronto Toronto, ON Canada; 7 Division of Emergency Medicine St. Joseph’s Healthcare Hamilton Hamilton, ON Canada; 8 Department of Medicine McMaster University Hamilton, ON Canada; 9 Dalla Lana School of Public Health University of Toronto Toronto, ON Canada; 10 North York General Hospital Toronto, ON Canada; 11 Ontario Health Toronto, ON Canada; 12 ICES Toronto, ON Canada; 13 Schwartz/Reisman Emergency Medicine Institute Sinai Health Toronto, ON Canada; 14 Division of Emergency Medicine Department of Family and Community Medicine, Temerty Faculty of Medicine University of Toronto Toronto, ON Canada

**Keywords:** virtual urgent care, health care expenditures, Canada, virtual care, economic evaluation, pilot program, pilot, Ontario, urgent care, care, emergency department, users, patient, patients, resources, resource allocation, policy decision, decision-making, policy

## Abstract

**Background:**

In 2020, the Ministry of Health (MoH) in Ontario, Canada, introduced a virtual urgent care (VUC) pilot program to provide alternative access to urgent care services and reduce the need for in-person emergency department (ED) visits for patients with low acuity health concerns.

**Objective:**

This study aims to compare the 30-day costs associated with VUC and in-person ED encounters from an MoH perspective.

**Methods:**

Using administrative data from Ontario (the most populous province of Canada), a population-based, matched cohort study of Ontarians who used VUC services from December 2020 to September 2021 was conducted. As it was expected that VUC and in-person ED users would be different, two cohorts of VUC users were defined: (1) those who were promptly referred to an ED by a VUC provider and subsequently presented to an ED within 72 hours (these patients were matched to in-person ED users with any discharge disposition) and (2) those seen by a VUC provider with no referral to an in-person ED (these patients were matched to patients who presented in-person to the ED and were discharged home by the ED physician). Bootstrap techniques were used to compare the 30-day mean costs of VUC (operational costs to set up the VUC program plus health care expenditures) versus in-person ED care (health care expenditures) from an MoH perspective. All costs are expressed in Canadian dollars (a currency exchange rate of CAD $1=US $0.76 is applicable).

**Results:**

We matched 2129 patients who presented to an ED within 72 hours of VUC referral and 14,179 patients seen by a VUC provider without a referral to an ED. Our matched populations represented 99% (2129/2150) of eligible VUC patients referred to the ED by their VUC provider and 98% (14,179/14,498) of eligible VUC patients not referred to the ED by their VUC provider. Compared to matched in-person ED patients, 30-day costs per patient were significantly higher for the cohort of VUC patients who presented to an ED within 72 hours of VUC referral ($2805 vs $2299; difference of $506, 95% CI $139-$885) and significantly lower for the VUC cohort of patients who did not require ED referral ($907 vs $1270; difference of $362, 95% CI 284-$446). Overall, the absolute 30-day costs associated with the 2 VUC cohorts were $18.9 million (ie, $6.0 million + $12.9 million) versus $22.9 million ($4.9 million + $18.0 million) for the 2 in-person ED cohorts.

**Conclusions:**

This costing evaluation supports the use of VUC as most complaints were addressed without referral to ED. Future research should evaluate targeted applications of VUC (eg, VUC models led by nurse practitioners or physician assistants with support from ED physicians) to inform future resource allocation and policy decisions.

## Introduction

Although virtual care has been available for several decades, it was not widely used before the COVID-19 pandemic. In Ontario, the most populous province in Canada (2021 population: 14.2 million [1]), the Ontario Telemedicine Network (OTN) was the only fully funded virtual care model prior to the COVID-19 pandemic. Historically, two-thirds of OTN use has been for mental health and substance use [2]. In the second quarter of 2019, OTN accounted for approximately 1.6% [3] of all ambulatory care visits. Following the first wave of the COVID-19 pandemic and the introduction of physician billing codes to support virtual care in Ontario, the percentage of ambulatory care visits in Ontario delivered by virtual care in the second quarter of 2020 has increased to 70.6% [3]. In parallel, between the pre–COVID-19 period (March 12 to July 29, 2019) and the COVID-19 period (March 11 to July 28, 2020), the number of in-person physician office visits decreased by approximately 80% [4].

In the fall of 2020, the Ontario Ministry of Health (MoH) introduced a virtual urgent care (VUC) pilot program to provide alternative access to urgent care services and reduce the need for in-person emergency department (ED) visits for patients with low acuity health concerns [5]. Hospitals interested in offering VUC services were invited to submit a funding application to Ontario Health, Ontario’s health care administrative agency. Following a review and feedback process, the MoH approved $4 million of operational funding to support 14 ED-led VUC programs in the 5 health regions of Ontario. As part of this funding, participating sites were expected to launch their VUC programs within 1 to 2 months of funding approval and participate in a mixed methods evaluation of the VUC pilot program to inform future policy and funding decisions. The characteristics of the VUC sites [6] and patient characteristics [7] are described elsewhere but are briefly summarized here. The 14 VUC sites included a mix of pediatric, northern, urban, academic, and community sites across Ontario. Since sites were responsible for the design and implementation of the VUC program to meet their local needs, models of care differed between sites (eg, clinical and operational governance, triage, technology, staffing, operating hours, marketing, and communications) and sites launched their VUC services at different start dates [6].

There were 22,278 VUC encounters between December 2020 and September 2021, and the median (IQR) duration of the virtual visit was 14 (11-18) minutes [7]. VUC users were more likely to be younger, female, English speaking, and have a postsecondary or higher education and a primary care physician. Most complaints were managed by the VUC provider without the need to refer the patient to an in-person ED visit [7]. Other results based on 1:1 propensity score matching (PSM) methods indicated that 30-day health care use (eg, hospitalization, ED, and physician visits) was similar between 2129 VUC users who presented to an ED within 72 hours of VUC referral and 2129 matched patients presenting to the ED in person [8]. VUC users not promptly referred to an ED (N=14,179) were more likely to have an ED visit within 72 hours, 7 days, and 30 days following the index VUC visit compared to matched in-person ED individuals [8]. However, 30-day hospital admissions were lower for the VUC group not promptly referred to the ED, but the length of hospital admission was longer than for patients presenting to the ED in person [8].

To inform future investments in VUC in Canada and elsewhere, here, we present the results from the costing analysis of the VUC pilot program that consider both funding provided by the MoH in Ontario to operate the VUC program and direct costs associated with health care resource use during the 30 days following each VUC visit. Our research hypotheses were that (1) VUC users promptly referred to an in-person ED by their VUC provider incur higher costs than matched in-person ED users with any type of discharge and (2) VUC users who were not referred to an in-person ED by their VUC provider incur lower 30-day costs than matched in-person ED users discharged home by the ED physician.

## Methods

### Study Design

We conducted [8] a population-based, matched cohort study of Ontarians who used VUC services provided by 12 of the 14 ED-led pilot sites from December 2020 to September 2021. Records from 2 sites were excluded as 1 site had a provider-to-provider VUC model (ie, physicians or nurses consulting with ED physicians) and the other site was delayed in launching the VUC program. These data sets were linked using unique encoded identifiers and analyzed at ICES (formerly the Institute for Clinical Evaluative Sciences). Patient-level VUC encounter data were linked to administrative health care databases (Multimedia Appendix 1) held at ICES to compare the 30-day costs of VUC versus in-person ED care from an MoH perspective. ICES data contain all health care services covered by the Ontario Health Insurance Plan, which include all hospital care, ED visits, hospital-based outpatient specialty clinics, physician visits for all residents of Ontario (40% of the Canadian population), and prescription drugs for residents aged 65 years and older and social assistance recipients. The analyses and reporting follow the STROBE (Strengthening the Reporting of Observational Studies in Epidemiology) guidelines [9].

### Ethical Considerations

The study protocol for the collection of VUC data was reviewed and approved by the Research Ethics Board at Sinai Health (21-003E), Toronto, Ontario, Canada. Individual resident consent was not required to access and use provincial health care records for this study, as it was conducted at ICES, an independent, not-for-profit health services research institute whose legal status under Ontario’s Personal Health Information Protection Act authorizes it to collect personal health information, without consent, for the purposes of analysis or compiling statistical information with respect to the management of, evaluation or monitoring of, the allocation of resources to or planning for all or part of the health system. All data analyses were performed at ICES, where the data are securely held in linked, coded (deidentified) form.

### Population

The study population included both pediatric and adult Ontario residents visiting 1 of the 12 included VUC pilot programs or any Ontario in-person ED from December 2020 to September 2021. Non-Ontario residents, individuals without a valid Ontario Health Insurance Plan number, and those who left without seeing a VUC provider were excluded from the analyses. We also excluded individuals who did not show up to an in-person ED within 72 hours after being promptly referred to the ED as it was deemed that an ED visit after 72 hours was less likely to be directly related to the initial visit issue (or at least the extent or severity of the issue at that time). As some individuals may have multiple VUC encounters, the first VUC encounter was considered the index VUC visit.

Since VUC users were expected to be different from patients who presented in person to the ED, VUC users were split into those who presented to an ED within 72 hours of VUC referral and those who saw a VUC provider with no referral to an in-person ED (“discharged home” by the VUC provider) [8]. Using propensity scoring matching methods, the first group of VUC users who presented to an ED within 72 hours of VUC referral was matched with a comparable group of individuals presenting in-person to an ED with any discharge disposition (eg, admitted, transferred, and discharged home). The second group of VUC users discharged home was matched with a comparable group of individuals presenting in person to an ED and discharged home by the ED physician. In addition, to generate comparable groups of VUC and in-person ED users based on their discharge disposition, this stratification allowed each matched group to have an equal opportunity to consume health care resources and incur costs.

### Primary and Secondary Outcomes

The primary outcome was 30-day MoH costs following the first VUC encounter or matched in-person ED encounter. Secondary outcomes were 30-day health care resource use (hospitalizations, ED visits, same-day surgeries, physician visits, outpatient publicly funded drugs, laboratory tests, and nonphysician visits) and associated health care expenditures.

### Statistical Analysis

To match our 2 subgroups of VUC patients with comparable patients who presented in person to an ED, we used a greedy nearest-neighbor 1:1 matching based on the index encounter date (±14 days), day (weekday vs weekend), and time of registration (day: 8 AM-8 PM; night: 8 PM-8 AM), presenting Canadian Emergency Department Information System complaint [10] and the logit of a propensity score. Canadian Emergency Department Information System includes 169 presenting complaints divided into 20 categories [10]. The variables included in the propensity score were age; sex; Statistics Canada Census neighborhood income quintile; urban or rural residence status; Ontario Marginalization Index [11] quintiles related to ethnic concentration, residential instability, material deprivation, and dependency; formal rostering with a family physician; the number of major ACG System Aggregated Diagnosis Groups derived from the Johns Hopkins ACG System version 10 (Health Services Research & Development Center, The Johns Hopkins University, Bloomberg School of Public Health) grouped as 0-4, 5-9, 10-14, 15+; medical conditions derived from validated administrative data algorithms (asthma, congestive heart failure, hypertension, chronic obstructive pulmonary disease, diabetes, and dementia); and the number of physician visits, ED visits, and hospitalizations in the 365 days preceding the index date. A caliper width of 0.2 of the SD of the logit of the propensity score was used. Balance in baseline covariates for each cohort was evaluated using standardized differences, with values less than 0.10 indicating that the groups were well matched [12].

Per-patient mean (SD) 30-day health care expenditures (eg, ED, physician, and hospitalization) expressed in 2020-2021 Canadian dollars (a currency exchange rate of CAD $1=US $0.76 is applicable) were calculated for each cohort using standardized costing algorithms for Ontario administrative health care data in Ontario [13]. The costing methodology uses a bottom-up or micro-costing approach to cost services at the individual level and costs represent amounts paid by the Ontario MoH [13]. Operational funding provided by the MoH to establish the VUC programs was added to the 30-day health care expenditures for the VUC cohort using site-specific information (ie, total operational funds received by each hospital divided by the total number of VUC visits to each hospital). Bootstrap techniques were used to generate 95% CIs associated with the difference in 30-day costs per patient between the VUC cohorts (operational funds plus health care expenditures) and matched in-person ED cohorts (health care expenditures). The same approach was used to analyze differences in secondary outcomes. In addition to the per-patient analyses, we present the total absolute costs for each cohort. All analyses were performed at ICES using linked, encoded data using SAS (version 9.4; SAS Institute).

## Results

### Study Population

There were 22,278 patient encounters in the pilot program, of which 19,595 patient VUC encounters were available for data linkage. This included 2931 (15%) VUC encounters that resulted in a referral to an in-person ED and 16,664 (85%) VUC encounters that did not result in a referral to an in-person ED. After applying our eligibility criteria, records from 2150 patients promptly referred to the ED and who presented to the ED within 72 hours (Multimedia Appendix 2) and records from 14,498 patients who were not referred to an in-person ED by their VUC provider (Multimedia Appendix 3) were available for the matched analyses. An additional 669 patients promptly referred to the ED by their VUC provider did not present to an ED within 72 hours and were excluded from the matched analysis as per our exclusion criteria. After 1:1 matching, 2129 patients who presented to the ED within 72 hours of a referral by a VUC provider and 14,179 patients seen by a VUC provider with no referral to an in-person ED were matched to an equal number of in-person ED individuals. Our matched VUC cohorts represented 99% (2129/2150) and 98% (14,179/14,498) of all eligible VUC patients, respectively.

### VUC Patients Who Were Promptly Referred to the ED and Who Presented to the ED Within 72 Hours and Matched In-Person ED Controls

Pediatric sites accounted for approximately 41% (876/2129) of the cohort of VUC users promptly referred to the ED and who presented to the ED within 72 hours of being referred by their VUC provider. The mean (SD) age of the VUC cohort was 29.1 (24.9) years, and 10% (222/2129) were aged 65 years or older. Approximately 61% (1296/2129) of VUC users were female, 96% (2052/2129) were living in urban areas, and 96% (2050/2129) were rostered to a primary care physician. Upon ED presentation, 80% of acuity scores corresponded to Canadian Triage and Acuity Scale levels 3 (urgent: 1242/2129, 58%), 4 (less urgent: 402/2129, 19%), and 5 (nonurgent: 109/2129, 5%). The most common medical conditions of the VUC cohort were asthma (424/2129, 20%), hypertension (346/2129, 16%), and diabetes (175/2129, 8%). The mean (SD) numbers of in-person ED visits and hospitalizations in the preceding 365 days for this VUC cohort promptly referred to the ED were 1.0 (3.0) and 0.3 (0.7), respectively. Table 1 presents the baseline characteristics of our matched VUC users who presented to an ED within 72 hours of VUC referral and in-person ED cohorts (n=2129 each), which were well-balanced.

The 30-day health care expenditures were similar between the 2 cohorts. However, the 30-day MoH costs per patient were relatively greater for the VUC cohort (mean $2805, SD $7026, including mean $163, SD $99 of operational funds and mean $ 2642, SD $7017 of health care expenditures) compared to the matched in-person ED cohort (mean $2299, SD $6174), resulting in a difference of $506 (95% CI $139-$885) per patient (Table 2).

**Table 1:** Baseline characteristics of patients referred to an ED^a^ by a virtual urgent care provider and who presented to the ED within 72 hours and matched in-person ED controls^b^.

Characteristics			Virtual urgent care (n=2129)		In-person ED care (n=2129)		Standardized difference^c^
Age (years), mean (SD)			29.1 (24.9)		28.2 (22.1)		0.04
**Age (years), n (%)**							
	<18	876 (41.2)		781 (36.7)		0.09	
	18-64	1031 (48.4)		1175 (55.2)		0.14	
	≥65	222 (10.4)		173 (8.1)		0.08	
Female, n (%)			1296 (60.9)		1322 (62.1)		0.03
Living in an urban setting, n (%)			2052 (96.4)		2043 (96)		0.02
**Neighborhood income quintile, n (%)**							
	High (quintiles 4-5)	976 (45.8)		958 (45)		0.02	
	Other	1153 (54.2)		1171 (55)		0.02	
**Acuity score,^d^ n (%)**							
	Resuscitation (CTAS^e^ 1)	11 (0.5)		11-15^f^		0.01	
	Emergent (CTAS 2)	365 (17.1)		423 (19.9)		0.07	
	Urgent (CTAS 3)	1242 (58.3)		1161 (54.5)		0.08	
	Less-urgent (CTAS 4)	402 (18.9)		417 (19.6)		0.02	
	Nonurgent (CTAS 5)	109 (5.1)		114 (5.3)		0.01	
	Unknown	0 (0.0)		1-5^f^		0.04	
**Selected medical conditions, n (%)**							
	Asthma	424 (19.9)		397 (18.7)		0.03	
	Congestive heart failure	60 (2.8)		48 (2.3)		0.04	
	Hypertension	346 (16.3)		287 (13.5)		0.08	
	Chronic obstructive pulmonary disease	39 (1.8)		44 (2.1)		0.02	
	Diabetes	175 (8.2)		167 (7.8)		0.01	
	Dementia	30 (1.4)		23 (1.1)		0.03	
**Johns Hopkins ACG System ADGs^g^, n (%)**							
	0-4	687 (32.3)		673 (31.6)		0.01	
	5-9	925 (43.5)		977 (45.9)		0.05	
	10-14	407 (19.1)		383 (18)		0.03	
	15+	110 (5.2)		96 (4.5)		0.03	
Rostered to a primary care physician, n (%)			2050 (96.3)		2021 (94.9)		0.07
Number of physician (GP^h^ and specialists) visits in preceding 365 days, mean (SD)			9.8 (13.4)		8.9 (11.6)		0.07
Number of ED visits in proceeding 365 days, mean (SD)			1.0 (3.0)		0.9 (1.7)		0.05
Number of hospitalizations in preceding 365 days, mean (SD)			0.3 (0.7)		0.3 (0.8)		0.00

^a^ED: emergency department.

^b^In addition, the cohorts were matched in function of the Ontario Marginalization (ONMARG) Ethnic Diversity quintile, ONMARG Residential Instability quintile, ONMARG Material Deprivation, and ONMARG Dependency quintile (data not shown).

^c^Standardized differences greater than 0.1 are generally considered meaningful.

^d^For the cohort of VUC users referred to an ED and who presented to the ED within 72 hours, CTAS was evaluated at the time of the ED visit.

^e^CTAS: Canadian Triage and Acuity Scale.

^f^Cells are suppressed to protect patient privacy.

^g^ADG: aggregated diagnosis group.

^h^GP: general practitioner.

**Table 2:** The 30-day costs per patient associated with patients referred to an ED^a^ by a virtual urgent care provider and who presented to the ED within 72 hours and matched in-person ED controls.

Costs and health care resource use	VUC^b^, mean (SD)	In-person ED care, mean (SD)	VUC versus in-person ED care, mean difference (95% CI)
Hospitalizations (CAD $)^c^	$1367.33 ($6071.73)	$1179.76 ($5119.87)	$187.57 (–$123.25 to $504.81)
Number of hospitalizations	0.2 (0.4)	0.1 (0.4)	0.0 (0.0 to 0.0)
Same-day surgeries (CAD $)	$41.67 ($328.62)	$26.49 ($226.84)	$15.18 (–$1.15 to $31.03)
Number of same-day surgeries	0.0 (0.2)	0.0 (0.2)	0.0 (0.0 to 0.0)
ED visits (CAD $)	$472.99 ($414.14)	$436.67 ($369.78)	$36.32 ($14.91 to $57.95)
Number of ED visits	1.3 (0.7)	1.3 (0.6)	0.0 (–0.0 to 0.1)
Outpatient publicly funded drugs (CAD $)	$53.96 ($344.63)	$62.61 ($357.82)	–$8.65 (–$30.47 to $11.69)
Number of claims	1.2 (5.1)	1.7 (9.5)	–0.5 (–0.9 to –0.1)
Index day (day 1) physician visits^d,e^ (CAD $)	$212.08 ($176.23)	$182.39 ($214.57)	$29.69 ($17.99 to $41.51)
Day 1-30 physician visits^f^ (CAD $)	$691.49 ($997.00)	$574.90 ($1033.88)	$116.59 ($58.44 to $175.07)
GP^g^ visits (CAD $)	$74.75 ($178.08)	$62.35 ($139.29)	$12.40 ($3.60 to $21.55)
Number of GP visits	1.2 (1.6)	0.9 (1.4)	0.3 (0.2 to 0.4)
Specialist visits (CAD $)	$390.31 ($842.63)	$339.00 ($883.46)	$51.31 ($4.57 to $102.53)
Number of specialist visits	1.5 (1.5)	0.7 (1.2)	0.8 (0.7 to 0.9)
Other costs (shadow billing, etc; CAD $)	$215.94 ($345.02)	$165.42 ($348.67)	$50.52 ($29.59 to $71.45)
Capitation costs (CAD $)	$14.54 ($18.26)	$18.39 ($16.40)	–$3.85 (–$4.77 to –$2.97)
Laboratory test (CAD $)	$8.84 ($34.94)	$7.09 ($26.04)	$1.75 ($0.09 to $3.62)
Number of tests	1.4 (4.4)	1.3 (4.5)	0.2 (–0.1 to 0.4)
Nonphysician visits (CAD $)	$1.65 ($36.36)	$1.04 ($8.42)	$0.61 (–$0.53 to $2.63)
Number of nonphysician visits	0.0 (0.3)	0.0 (0.3)	0.0 (–0.0 to 0.0)
Total health care expenditures (CAD $)	$2641.97 ($7016.61)	$2298.82 ($6173.60)	$343.15 (–$23.54 to 719.88)
Operating funds (CAD $)	$163.24 ($98.64)	N/A^h^	N/A
Total costs (CAD $)	$2805.21 ($7,026.03)	$2298.82 ($6173.60)	$506.39 ($139.10 to $885.16)

^a^ED: emergency department.

^b^VUC: virtual urgent care.

^c^A currency exchange rate of CAD $1=US $0.76 is applicable.

^d^Due to the absence of the exact time of the billings, the “index day physician” visits include all physician visits (eg, general practitioners and specialists) that occurred during the index day (day 1).

^e^The costs associated with physician visits during the index day are included in the 30-day costs associated with physician visits (days 1-30).

^f^Physician costs are composed of the prices and quantity of each service billed by physicians to Ontario Health Insurance Plan, as well as shadow billing costs and capitation costs.

^g^GP: general practitioner.

^h^N/A: not applicable.

### VUC Patients Not Referred to an In-Person ED and Matched In-Person ED Controls

The baseline characteristics of the VUC cohort not promptly referred to an in-person ED by their VUC provider were similar to VUC users promptly referred to the ED by their VUC provider. Table 3 presents the baseline characteristics of our matched VUC and in-person ED cohorts (n=14,179 each), which were well balanced.

The 30-day mean (SD) MoH health care expenditures per patient were lower with VUC (mean $758, SD $3129) than in-person ED care (mean $1270, SD $3846; *P*<.001), resulting in a difference of $511 (95% CI $434-$595) per patient in favor of VUC. More than 90% ($480/$511) of this cost difference was explained by a significant reduction in 30-day ED costs ($237/$511, 46% of total cost reduction) and physician visits ($243/$511, 47%). After adding the VUC operational funding (mean $149, SD $95 per VUC user), VUC remained less expensive than in-person ED care. The details are provided in Table 4.

**Table 3:** Baseline characteristics of patients seen by a virtual urgent care provider with no referral to an in-person ED^a^ and matched in-person ED controls^b^.

Characteristics			Virtual urgent care (n=14,179)		In-person ED care (n=14,179)		Standardized difference^c^
Age (years), mean (SD)			28.1 (23.5)		27.6 (22.5)		0.02
**Age (years), n (%)**							
	<18	5640 (39.8)		5485 (38.7)		0.02	
	18-64	7386 (52.1)		7633 (53.8)		0.03	
	≥65	1153 (8.1)		1061 (7.5)		0.02	
Female, n (%)			8482 (59.8)		8704 (61.4)		0.03
Living in an urban setting, n (%)			13,533 (95.4)		13,330 (94)		0.06
**Neighborhood income quintile, n (%) **							
	High (quintiles 4-5)	6210 (43.9)		6202 (43.7)		0.00	
	Other	7969 (56.2)		7977 (56.3)		0.00	
**Selected medical conditions, n (%)**							
	Asthma	2778 (19.6)		2684 (18.9)		0.02	
	Congestive heart failure	232 (1.6)		252 (1.8)		0.01	
	Hypertension	1939 (13.7)		1801 (12.7)		0.03	
	Chronic obstructive pulmonary disease	223 (1.6)		247 (1.7)		0.01	
	Diabetes	1051 (7.4)		1161 (8.2)		0.03	
	Dementia	133 (0.9)		83 (0.6)		0.04	
**Johns Hopkins ADGs^d^, n (%)**							
	0-4	4777 (33.7)		4527 (31.9)		0.04	
	5-9	6427 (45.3)		6617 (46.7)		0.03	
	10-14	2460 (17.4)		2468 (17.4)		0.00	
	≥15	515 (3.6)		567 (4)		0.02	
Rostered to a primary care physician, n (%)			13,745 (96.9)		13,708 (96.7)		0.01
Number of physician (GP^e^ and specialists) visits in the preceding 365 days, mean (SD)			8.5 (10.8)		8.4 (11.2)		0.01
Number of ED visits in the proceeding 365 days, mean (SD)			0.9 (2.3)		1.0 (1.7)		0.03
Number of hospitalizations in the preceding 365 days, mean (SD)			0.2 (0.7)		0.2 (0.7)		0.03

^a^ED: emergency department.

^b^In addition, the cohorts were matched in function of the Ontario Marginalization (ONMARG) Ethnic Diversity quintile, ONMARG Residential Instability quintile, ONMARG Material Deprivation, and ONMARG Dependency quintile (data not shown).

^c^Standardized differences greater than 0.1 are generally considered meaningful.

^d^ADG: aggregated diagnosis groups.

^e^GP: general practitioner.

**Table 4:** The 30-day costs per patient associated with patients seen by a VUC^a^ provider with no referral to an in-person ED^b^ and matched in-person ED controls.

Costs and health care resource use	VUC, mean (SD)	In-person ED care, mean (SD)	VUC versus in-person ED care, mean difference (95% CI)
Hospitalizations (CAD $^c^)	$274.70 ($2485.70)	$356.64 ($3076.53)	–$81.94 (–$150.59 to -$19.08)
Number of hospitalizations	0.0 (0.2)	0.0 (0.2)	–0.0 (–0.0 to –0.0)
Same-day surgeries (CAD $)	$23.96 ($252.67)	$47.22 ($367.97)	–$23.26 (–$30.41 to –$15.81)
Number of same-day surgeries	0.0 (0.1)	0.0 (0.2)	–0.0 (–0.0 to –0.0)
ED visits (CAD $)	$127.00 ($288.33)	$364.42 ($356.44)	–$237.42 (–$244.24 to –$230.58)
Number of ED visits	0.4 (0.8)	1.3 (0.8)	–0.9 (–0.9 to –0.9)
Outpatient publicly funded drugs (CAD $)	$45.52 ($394.42)	$65.13 ($445.68)	–$19.61 (–$29.29 to –$10.27)
Number of claims	1.2 (6.0)	1.5 (6.5)	–0.3 (–0.5 to –0.2)
Index day (day 1) physician visits^d,e^ (CAD $)	$54.67 ($73.88)	$152.65 ($136.93)	–97.98 (–$100.62 to –$95.59)
Day 1-30 physician visits^f^ (CAD $)	$272.65 ($601.14)	$417.20 ($724.25)	–144.55 (–$159.72 to –$129.64)
GP^g^ visits (CAD $)	$50.33 ($136.31)	$54.85 ($153.31)	–$4.52 (–$7.69 to –$1.35)
Number of GP visits	1.2 (1.6)	0.9 (1.4)	0.3 (0.2 to 0.3)
Specialist visits (CAD $)	$151.50 ($501.17)	$212.01 ($601.28)	–$60.51 (–$72.95 to –$47.99)
Number of specialist visits	1.0 (1.3)	0.7 (1.3)	0.3 (0.3 to 0.4)
Other costs (CAD $)	$62.00 ($159.04)	$142.06 ($219.03)	–$80.06 (–$84.61 to –$75.50)
Capitation costs (CAD $)	$14.59 ($17.31)	$19.09 ($15.89)	–$4.50 (–$4.88 to –$4.13)
Laboratory test (CAD $)	$7.86 ($28.91)	$7.03 ($26.68)	$0.83 ($0.17 to $1.45)
Number of tests	1.5 (4.7)	1.3 (4.3)	0.2 (0.1 to 0.3)
Nonphysician visits (CAD $)	$0.98 ($9.00)	$1.24 ($16.74)	–$0.26 (–$0.59 to $0.03)
Number of nonphysician visits	0.0 (0.3)	0.0 (0.3)	–0.0 (–0.0 to 0.0)
Total health care expenditures (CAD $)	$758.37 ($3128.81)	$1269.70 ($3846.26)	–$511.33 (–$595.26 to -$433.55)
Operating funds (CAD $)	$149.12 ($94.91)	N/A^h^	N/A
Total costs (CAD $)	$907.49 ($3133.39)	$1269.70 ($3846.26)	–$362.21 (–$446.26 to -$284.24)

^a^VUC: virtual urgent care.

^b^ED: emergency department.

^c^A currency exchange rate of CAD $1=US $0.76 is applicable.

^d^Due to the absence of the exact time of the billings, the “index day physician” visits include all physician visits (eg, GP and specialists) that occurred during the index day (day 1).

^e^The costs associated with physician visits during the index day are included in the 30-day costs associated with physician visits (days 1-30).

^f^Physician costs are composed of the prices and quantity of each service billed by physicians to Ontario Health Insurance Plan, as well as shadow billing costs and capitation costs.

^g^GP: general practitioner.

^h^N/A: not applicable.

### Total Absolute Costs for the VUC and In-Person ED Cohorts

At the cohort level, the absolute 30-day cost associated with the cohort of VUC users who presented to an ED within 72 hours of VUC referral and matched in-person ED patients (n=2129 each) were $6.0 million and $4.9 million, respectively. For the cohort of VUC users not referred to the ED by their VUC provider and the matched cohort of individuals attending in-person to the ED and discharged home by their ED physician (n=14,179 each), the absolute 30-day costs were $12.9 million and $18.0 million, respectively. Overall, the absolute 30-day costs associated with the two VUC cohorts were $18.9 million (ie, $6.0 million + $12.9 million) versus $22.9 million ($4.9 million + $18.0 million) for the 2 in-person ED cohorts.

## Discussion

### Principal Findings

The data from this Ontario VUC pilot program indicate that approximately 15% (2931/19,595) of VUC encounters were promptly referred to an in-person ED. Our costing analysis of 2129 patients who presented to the ED within 72 hours of a referral by a VUC provider indicated that there were no statistically significant differences in 30-day health care expenditures when compared to matched in-person ED patients. However, when the operational costs to set up the VUC programs were considered, the VUC cohort promptly referred to the ED incurred significantly higher costs. Due to differences in ED and physician costs, the 30-day costs of the cohort of VUC users not promptly referred to the ED were significantly lower than those of a matched cohort of in-person ED users.

These findings are important for several reasons. First, the data from the Ontario VUC pilot program evaluation suggest that VUC models may represent a good investment of government funding, especially if the operational costs to set up and maintain VUC models decrease over time with subsequent implementation efficiencies. However, considering that most VUC complaints were dealt with by the VUC provider with no referral to the ED, other models of VUC where nurse practitioners, physician assistants, or family physicians provide first-line VUC instead of emergency physicians may be more attractive, especially in the context of current severe ED workforce shortages. Finally, this study proposes a costing methodology to deal with both the heterogeneity between VUC users and non-VUC users, which allows more meaningful comparisons.

### Comparison With Prior Work

To our knowledge, this study is the first to evaluate the economic costs of VUC in Canada. Although several US studies have shown telemedicine or VUC for low acuity patients save costs from a health system perspective [14-17] or patient perspective [18], these results may not be generalizable to the Canadian health care system and these studies predate the COVID-19 pandemic. While recent Canadian data from April 2021 to March 2022 indicate the number of ED visits has almost returned to pre–COVID-19 levels [19], EDs across Canada continue to experience great pressures due to severe staffing shortages [20]. In this sense, VUC models could serve to complement in-person ED care.

### Strengths and Limitations

Compared to previous economic studies of virtual care, this study has several strengths. First, we had access to linked records of all Ontarians who used VUC services across the VUC pilot programs, which allowed us to document the 30-day health care use and costs. Second, we were able to match 98% (16,308/16,648) of all eligible VUC users. Third, we separated the VUC users into 2 cohorts based on their discharge status following the VUC encounter (“discharged home” or “referred to in-person ED”) and we matched each VUC cohort with comparable in-person ED users (eg, the VUC users not referred to an in-person ED by their VUC provider were matched to comparable in-person ED individuals discharged home by their ED physician), which allowed us to decrease the heterogeneity between patients using VUC and in-person ED care. Finally, we used PSM methods and bootstrap techniques to compare matched VUC and in-person ED cohorts and included VUC infrastructure costs in our cost calculations.

This study also has important limitations as patients who choose VUC may be different from patients who attend the ED in person. Since the decision to use VUC versus attending an ED in person is influenced by multiple factors, many of which are not captured in administrative data (eg, self-perceived symptom severity at the time of decision, time and costs to travel to the ED, and satisfaction with prior ED services), comparing users of VUC with those who attend in-person ED is methodologically challenging. To deal with this important limitation and the heterogeneity between VUC users and between VUC and in-person ED users, we split and matched our VUC users by discharge disposition (eg, VUC presenting to the ED within 72 hours of a VUC referral or discharged home) and used PSM methods to compare with in-person ED patients. Despite matching on many measured confounders, important potential confounders were not available to us and the risk for potential unmeasured confounding remains. For example, the pediatric population and the senior population may require assistance with travel to the ED, which may influence decisions to use VUC rather than attend the ED in person. Although we matched the presenting complaint leading to the encounter, we were not able to match on acuity scores for the cohort of VUC users not referred to the ED by their VUC provider. In the VUC pilot, nearly 80% of VUC users screened themselves using a symptom checklist [7]. As such, caution should be expressed when interpreting the results of the matched analyses for the VUC cohort not referred to ED. We also did not know if VUC users would have presented in person to the ED in the absence of the VUC pilot program. They could have gone, for example, to an outpatient urgent care or walk-in clinic. However, compared to other jurisdictions, urgent care clinics are limited in Ontario. For example, while there are approximately 140 hospitals in Ontario, there are less than 20 urgent care centers in Ontario [21]. While there are more walk-in clinics in Ontario than urgent care centers, walk-in clinics do not have ED physicians on staff, and therefore, walk-in clinics may not be an appropriate comparator. In addition, as opposed to hospitalization or ED visits, reporting outpatient clinic data is not mandatory in Ontario [22]. As such, we did not have access to these data to create a control group of individuals attending urgent care outpatient clinics.

While we had access to several administrative databases, privately funded drug expenditures were not included in our analyses nor did we include patients’ time (including economic impact and opportunity costs) or out-of-pocket expenditures (eg, parking and travel), which may have been avoided by the availability of VUC. As this was a pilot project, the population who would use VUC should it become universally available across Ontario may be different. However, we believe the mix of the VUC sites participating in the pilot program is broadly representative of the breadth of ED settings in Ontario. There may be differences in 30-day health care use and costs between models of care (eg, nurse triage vs self-screening), populations (pediatrics vs adults), or settings (community vs academic sites; rural vs urban), which we did not examine. Due to data limitations, we were not able to isolate the physician costs associated with the VUC or in-person ED encounter. Finally, the MoH operational funds used in our calculations may represent an underestimation of the true costs of setting up VUC programs as it did not include any in-kind support provided by the sites. While the operational costs to set up and maintain VUC models may decrease with efficiencies in implementation, the cost of providing VUC is likely to change if different models of care are implemented (eg, nurse practitioners or physician assistants as first VUC contact before escalating to the ED physician if needed) or if the VUC technology becomes more expensive. Generalizations on health system costs within this study are limited to acute and subacute patient outcomes treated within the 30-day window of this study, and results may not be generalizable to other time periods or health care settings. Finally, due to the lack of data, our analyses were limited to costs and did not include economic outcomes (eg, quality-adjusted life-years). Therefore, the cost-effectiveness of the VUC program is unknown and this is left for future research.

### Conclusions

This costing analysis supports the use of VUC for low acuity presenting complaints as most patient concerns were addressed without an in-person referral to the ED. However, additional research should evaluate alternative models of VUC to inform future resource allocation and policy decisions.

## Appendix

Multimedia Appendix 1 ICES databases.

Multimedia Appendix 2 Cohort selection of virtual urgent care (VUC) patients referred to the emergency department after their VUC encounter and before 1:1 matching.

Multimedia Appendix 3 Cohort selection of virtual urgent care (VUC) patients not referred to an in-person emergency department after their VUC encounter and before 1:1 matching.

## Abbreviations

ED: emergency department
MoH: Ministry of Health
OTN: Ontario Telemedicine Network
PSM: propensity score matching
STROBE: Strengthening the Reporting of Observational Studies in Epidemiology
VUC: virtual urgent care

## Data Availability

The data sets analyzed during this study are not publicly available due to legal data sharing agreements between ICES and data providers. However, confidential access may be granted to those who meet prespecified ICES criteria by contacting the corresponding author.
