# Supplementary material for: Cost Evaluation of the Ontario Virtual Urgent Care Pilot Program: Population-Based, Matched Cohort Study
Source: J Med Internet Res. 2024 Jul 15;26:e50483. doi: 10.2196/50483 (PMC11287093; doi:10.2196/50483)
Supplement: Multimedia Appendix 1 [file jmir_v26i1e50483_app1.docx]

| **Type of Healthcare Expenditures and Utilization** | **Name of Database** |
| --- | --- |
| Hospitalizations | Canadian Institute for Health Information (CIHI) - Discharge Abstract Database |
| Emergency departments, cancer care clinics, dialysis clinics | National Ambulatory Care Reporting System (NACRS) |
| Same day surgeries | CIHI – Same Day Surgery (SDS) database |
| Physician (general practitioner and specialists) and non-physician (e.g., optometrist) billings, laboratory tests billings, | Ontario Health Insurance Plan (OHIP) database |
| Prescription drugs | Ontario Drug Benefit Program (ODB) |
| Mortality | Ontario Registered Persons Database (RPDB) |
