# Supplementary material for: Cost Evaluation of the Ontario Virtual Urgent Care Pilot Program: Population-Based, Matched Cohort Study
Source: J Med Internet Res. 2024 Jul 15;26:e50483. doi: 10.2196/50483 (PMC11287093; doi:10.2196/50483)
Supplement: Multimedia Appendix 2 [file jmir_v26i1e50483_app2.docx]

| **Step** | **Criteria** | **Description** | **Records remaining** |
| --- | --- | --- | --- |
| 1 | Inclusion | All records in the Provincial VUC Pilot dataset | 22,278 |
| 2 | Exclusion | Excluding records with an invalid IKN (unable to link to ICES data) | 21,423 |
| 3 | Exclusion | Excluding records with no date/time available | 21,360 |
| 4 | Exclusion | Excluding records with invalid or incomplete data |  |
| 5 | Exclusion | Excluding non-Ontario residents | 21,319 |
| 6 | Exclusion | Excluding duplicate records | 21,078 |
| 7 | Exclusion | Excluding OHIP ineligible patients | 20,673 |
| 8 | Exclusion | Excluding records where patient left without being seen | 19,838 |
| 9 | Exclusion | Excluding records belonging to the Cohort of patients *not* promptly referred to the ED | 2,963 |
| 10 | Exclusion | Excluding records who did not show up to an in-person ED within 72hrs after being promptly referred | 2,279 |
| 11 | Exclusion | Excluding records belonging to two sites^a^ | 2,250 |
| 12 | Exclusion | Excluding records not possible to match (i.e., missing data required for PS calculation, complaint that doesn't match CEDIS) | 2,224 |
| 13 | Exclusion | Excluding multiple records belonging to the same individual | 2,150 |
|  |  | **Total** | **2,150** |

Notations: IKN: ICES Key Number; ED; emergency department; PS: propensity scoring; CEDIS: Canadian Emergency Department Information System

a: Records from two sites were excluded as one site had a provider-to-provider VUC model (i.e., physicians/nurses consulting with ED physicians) and the other site was delayed in launching the VUC program.
